# Establishment of CMab-43, a Sensitive and Specific Anti-CD133 Monoclonal Antibody, for Immunohistochemistry

**DOI:** 10.1089/mab.2017.0031

**Published:** 2017-10-01

**Authors:** Shunsuke Itai, Yuki Fujii, Takuro Nakamura, Yao-Wen Chang, Miyuki Yanaka, Noriko Saidoh, Saori Handa, Hiroyoshi Suzuki, Hiroyuki Harada, Shinji Yamada, Mika K. Kaneko, Yukinari Kato

**Affiliations:** ^1^ Department of Antibody Drug Development, Tohoku University Graduate School of Medicine, Sendai, Japan.; ^2^ Department of Oral and Maxillofacial Surgery, Graduate School of Medical and Dental Sciences, Tokyo Medical and Dental University, Tokyo, Japan.; ^3^ Department of Regional Innovation, Tohoku University Graduate School of Medicine, Sendai, Japan.; ^4^ Department of Pathology and Laboratory Medicine, Sendai Medical Center, Sendai, Japan.; ^5^ New Industry Creation Hatchery Center, Tohoku University, Sendai, Japan.

**Keywords:** CD133, monoclonal antibody, immunohistochemistry, colon cancer

## Abstract

CD133, also known as prominin-1, was first described as a cell surface marker on early progenitor and hematopoietic stem cells. It is a five-domain transmembrane protein composed of an N-terminal extracellular tail, two small cytoplasmic loops, two large extracellular loops containing seven potential glycosylation sites, and a short C-terminal intracellular tail. CD133 has been used as a marker to identify cancer stem cells derived from primary solid tumors and as a prognostic marker of gliomas. Herein, we developed novel anti-CD133 monoclonal antibodies (mAbs) and characterized their efficacy in flow cytometry, Western blot, and immunohistochemical analyses. We expressed the full length of CD133 in LN229 glioblastoma cells, immunized mice with LN229/CD133 cells, and performed the first screening using flow cytometry. After limiting dilution, we established 100 anti-CD133 mAbs, reacting with LN229/CD133 cells but not with LN229 cells. Subsequently, we performed the second and third screening with Western blot and immunohistochemical analyses, respectively. Among 100 mAbs, 11 strongly reacted with CD133 in Western blot analysis. One of 11 clones, CMab-43 (IgG_2a_, kappa), showed a sensitive and specific reaction against colon cancer cells, warranting the use of CMab-43 in detecting CD133 in pathological analyses of CD133-expressing cancers.

## Introduction

Cancer stem cells (CSCs) share many of the properties of non-neoplastic stem cells. They are also characterized by extensive proliferation, self-renewal, invasion, metastasis, and drug resistance.^(1–3)^ Side-population cells and several protein markers specific to CSCs have been developed to isolate CSCs from cancer tissues and to investigate the CSC properties in cancer tissues.^(4)^ These CSC markers include CD133 and CD44.^(1,5–12)^

CD133, also known as prominin-1, was first described as a cell surface marker on hematopoietic stem cells.^(13)^ It is a five-transmembrane glycoprotein composed of an N-terminal extracellular tail, two small cytoplasmic loops, two large extracellular loops containing several potential glycosylation sites, and a short C-terminal intracellular tail.^(14)^ CD133 has been used as a marker to identify CSCs derived from primary solid tumors.^(1)^ Its expression is also used as a prognostic marker of gliomas.^(15)^

Herein, we produced sensitive and specific monoclonal antibodies (mAbs) against CD133, which can be used for flow cytometry, Western blot, and immunohistochemical analysis.

## Materials and Methods

### Cell lines

LN229, HCT-116, Chinese hamster ovary (CHO)-K1, Caco-2, and P3X63Ag8U.1 (P3U1) cell lines were obtained from the American Type Culture Collection (ATCC, Manassas, VA). LN229/CD133 and CHO/CD133 were produced by transfecting pCAG/PA-CD133-RAP-MAP into LN229 and CHO-K1 cells using Neon transfection system (Thermo Fisher Scientific, Inc., Waltham, MA) and Lipofectamine LTX (Thermo Fisher Scientific, Inc.), respectively. A few days after transfection, PA tag-positive cells^(16)^ were sorted using a cell sorter (SH800; Sony Corp., Tokyo, Japan). CHO-K1, CHO/CD133, and P3U1 cell lines were cultured in RPMI 1640 medium (Nacalai Tesque, Inc., Kyoto, Japan), and LN229, LN229/CD133, HCT-116, and Caco-2 cell lines were cultured in Dulbecco's modified Eagle's medium (DMEM) (Nacalai Tesque, Inc.), supplemented with 10% heat-inactivated fetal bovine serum (Thermo Fisher Scientific, Inc.), 100 units/mL of penicillin, 100 μg/mL of streptomycin, and 25 μg/mL of amphotericin B (Nacalai Tesque, Inc.) at 37°C in a humidified atmosphere containing 5% CO_2_ and 95% air.

### Production of hybridoma

Female 4-week-old BALB/c mice were purchased from CLEA Japan (Tokyo, Japan). Animals were housed under specific pathogen-free conditions. The Animal Care and Use Committee of Tohoku University approved all of the animal experiments described herein.

Mice were immunized using intraperitoneal (i.p.) injections of LN229/CD133 cells together with Imject Alum (Thermo Fisher Scientific, Inc.). After several additional immunizations, a booster injection of LN229/CD133 cells was intraperitoneally administered 2 days before harvesting spleen cells. Spleen cells were then fused with P3U1 cells using PEG1500 (Roche Diagnostics, Indianapolis, IN) or GenomONE-CF (Ishihara Sangyo Kaisha, Ltd., Osaka, Japan). The resulting hybridomas were grown in RPMI medium supplemented with hypoxanthine, aminopterin, and thymidine selection medium supplement (Thermo Fisher Scientific, Inc.).

Culture supernatants were screened using flow cytometry (first screening), Western blot (second screening), and immunohistochemical analyses (third screening). MAbs were purified from supernatants of hybridomas cultured in Hybridoma-SFM medium (Thermo Fisher Scientific, Inc.) using Protein G Sepharose 4 Fast Flow (GE Healthcare United Kingdom, Ltd., Buckinghamshire, England).

### Flow cytometry

Cells were harvested by brief exposure to 0.25% trypsin/1 mM ethylenediaminetetraacetic acid (EDTA) (Nacalai Tesque, Inc.). After washing with 0.1% bovine serum albumin (BSA)/phosphate buffered saline (PBS), the cells were treated with 1 μg/mL of anti-CD133 mAb (clone CMab-43) for 30 min at 4°C and then with Alexa Fluor 488-conjugated antimouse IgG (1:1000; Cell Signaling Technology, Inc., Danvers, MA). Fluorescence data were collected using EC800 or SA3800 Cell Analyzers (Sony Corp.).

### Western blot analysis

Cell lysates (10 μg) were boiled in sodium dodecyl sulfate sample buffer (Nacalai Tesque, Inc.) and proteins were then electrophoresed on 5%–20% polyacrylamide gels (Wako Pure Chemical Industries, Ltd., Osaka, Japan) and were transferred onto polyvinylidene difluoride (PVDF) membranes (Merck KGaA, Darmstadt, Germany). After blocking with 4% skim milk (Nacalai Tesque, Inc.), membranes were incubated with 1 μg/mL of anti-CD133 mAb (clone CMab-43) and anti-β-actin (clone AC-15; Sigma-Aldrich Corp., St. Louis, MO), and then with peroxidase-conjugated antimouse IgG (1:1000 diluted; Agilent Technologies, Inc., Santa Clara, CA), and were finally developed using ImmunoStar LD (Wako Pure Chemical Industries, Ltd.) using a Sayaca-Imager (DRC Co., Ltd., Tokyo, Japan).

### Determination of binding affinity using flow cytometry

LN229/CD133 and Caco-2 cells (2 × 10^5^) were suspended in 100 μL of serially diluted mAbs (6–100 μg/mL), and Alexa Fluor 488-conjugated antimouse IgG (1:200; Cell Signaling Technology, Inc.) was then added. Fluorescence data were collected using a cell analyzer (EC800; Sony Corp.). The dissociation constants (*K*_D_) were calculated by fitting the binding isotherms using the built-in one-site binding models in GraphPad PRISM 6 (GraphPad software, Inc., La Jolla, CA).

### Immunohistochemical analyses

Colon cancer tissues were purchased from U.S. Biomax, Inc. (Rockville, MD). Histological sections of 4-μm thickness were deparaffinized in xylene and were then rehydrated and autoclaved in citrate buffer (pH 6.0; Agilent Technologies, Inc.) for 20 minutes. Sections were then incubated with 1 μg/mL of CMab-43 for 1 hour at room temperature and were then treated using an Envision+ kit (Agilent Technologies, Inc.) for 30 minutes. Color was developed using 3,3-diaminobenzidine tetrahydrochloride (Agilent Technologies, Inc.) for 2 minutes, and sections were then counterstained with hematoxylin (Wako Pure Chemical Industries, Ltd.).

## Results

In this study, we immunized mice with LN229/CD133 cells followed by a booster i.p. injection of LN229/CD133 cells. Culture supernatants were screened using flow cytometry to assess reactions with LN229 and LN229/CD133 cells. A total of 100 clones were generated after limiting dilution. Subsequently, mAbs were selected according to their signal efficacy on Western blot analysis. These analyses identified 79 of the 100 clones that were able to detect CD133, of which 11 exerted a strong and specific reaction. The 11 clones selected by Western blot analysis were further tested, and immunohistochemical analysis demonstrated that 4 clones showed strong staining, 4 showed moderate staining, and 3 showed no staining against colon cancer tissues. Three of the four clones that showed a strong staining against colon cancer tissues were determined to be of the IgG_1_ subclass; the fourth clone, CMab-43, was of the IgG_2a_ subclass and was selected for the subsequent study.

**FIG. 1.**
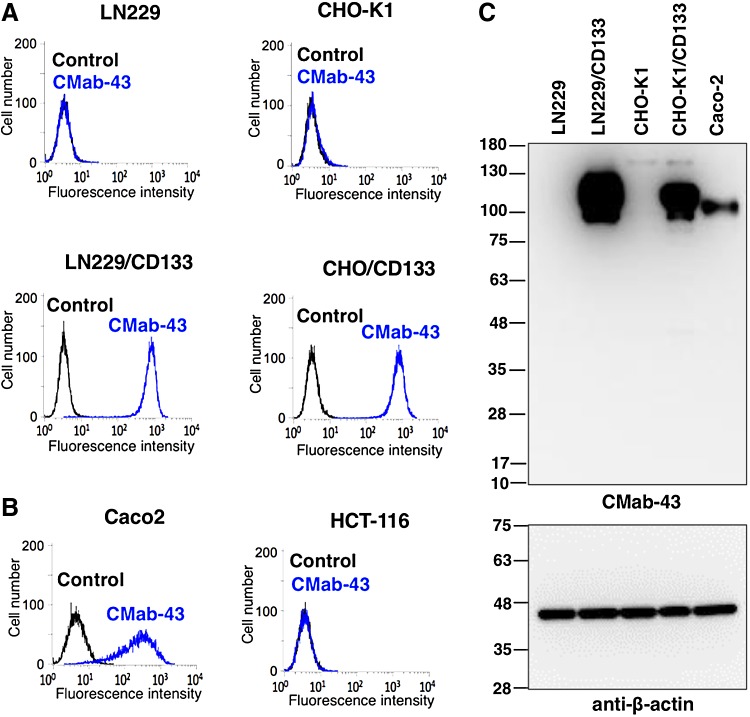
Characterization of CMab-43. **(A, B)** Flow cytometry with CMab-43; cells were treated with 1 μg/mL of CMab-43 followed by Alexa Fluor 488-conjugated antimouse IgG; black line, negative control. **(C)** Western blot using CMab-43; cell lysates (10 μg) were electrophoresed and proteins were transferred onto polyvinylidene difluoride (PVDF) membranes. After blocking, membranes were incubated with 1 μg/mL of CMab-43 or anti-β-actin (AC-15) and then incubated with peroxidase-conjugated antimouse IgG.

Flow cytometry demonstrated that CMab-43 reacts with LN229/CD133 cells but not with LN229 brain tumor cells (Fig. 1A). CMab-43 reacted with CHO/CD133 cells but not with the CHO-K1 parental cells, indicating that CMab-43 is specific for CD133 (Fig. 1A). CMab-43 also recognized endogenous CD133 in Caco-2 colon cancer cells, but did not react with HCT-116 colon cancer cells (Fig. 1B). On Western blot analyses against LN229, LN229/CD133, CHO-K1, CHO/CD133, and Caco-2 cells, CMab-43 detected a strong signal at ∼100 kDa in LN229/CD133 and CHO/CD133, and did a moderate signal in Caco-2 cells, indicating that CMab-43 is very useful in Western blot analyses (Fig. 1C). The broad band revealed by CMab-43 is likely due to various glycosylation forms of CD133, known to be highly glycosylated.^(17)^

**FIG. 2.**
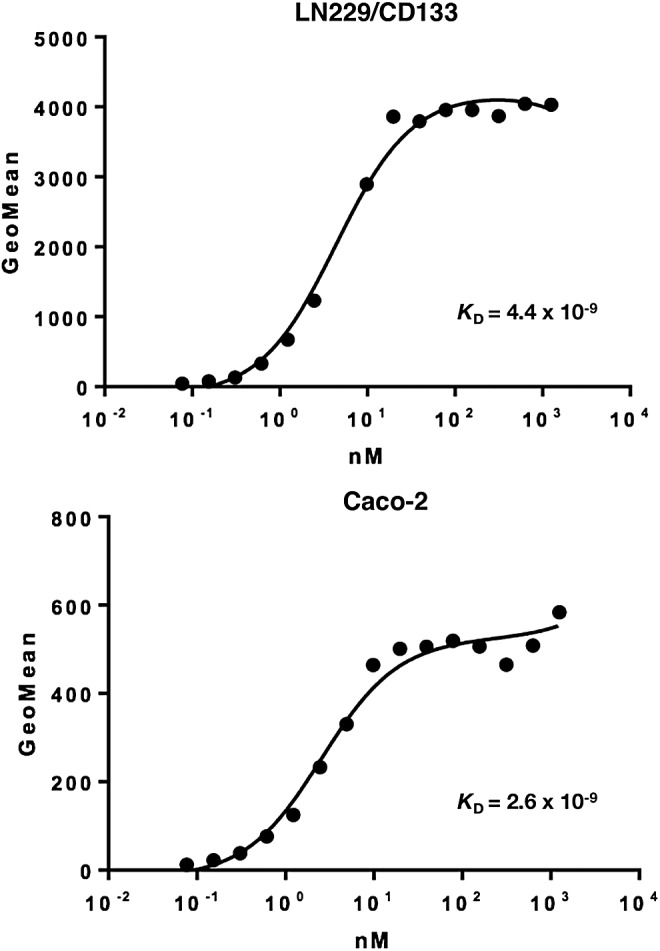
Binding affinity of CMab-43 was determined using flow cytometry. LN229/CD133 and Caco-2 cells were suspended in 100 μL of serially diluted CMab-43 (6 ng/mL–100 μg/mL), and secondary antimouse IgG was then added. Fluorescence data were collected using a cell analyzer.

We further determined the binding affinity of CMab-43 for Caco-2 and LN229/CD133 cells using flow cytometry (Fig. 2). The calculated *K*_D_ values for CMab-43 against LN229/CD133 and Caco-2 cells are 4.4 × 10^−9^ M and 2.6 × 10^−9^ M, respectively, indicating a high affinity for CD133-expressing cell lines.

**FIG. 3.**
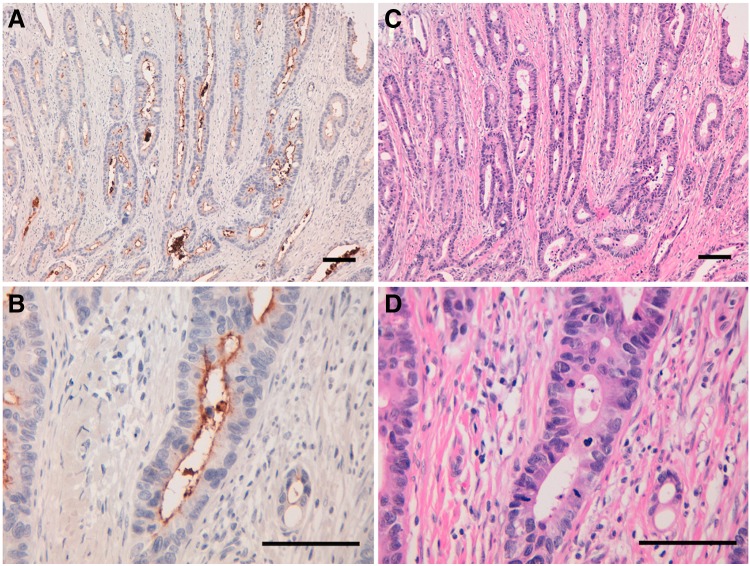
Immunohistochemical analysis by CMab-43 against colon cancer tissues. **(A, B)** Sections were incubated with 1 μg/mL of CMab-43 for 1 hour at room temperature followed by treatment with Envision+ kit for 30 minutes. Color was developed using 3,3-diaminobenzidine tetrahydrochloride for 2 minutes, and sections were then counterstained with hematoxylin. **(C, D)** Hematoxylin & eosin staining; scale bar = 100 μm.

We investigated the immunohistochemical utility of CMab-43 in human colon cancers as high CD133 expression was observed in Caco-2 colon cancer cell lines (Fig. 1C). Recent studies have shown CD133 expression with luminal staining.^(18–20)^ Staining of intraglandular debris was also observed in colorectal cancers. As shown in Figure 3 and Supplementary Figure 1, CMab-43 showed a luminal membrane expression pattern, and intraglandular debris was also stained. Normal mucosae were not positive for CMab-43 staining (Supplementary Fig. S2). CMab-43 stained 5/6 cases (83.3%) of colon adenocarcinomas (Supplementary Fig. S2 and Supplementary Table S1), indicating that CMab-43 is useful for immunohistochemical analysis.

## Discussion

Recently, we produced antipodoplanin (PDPN) cancer-specific mAbs clone LpMab-2^(21,22)^ and LpMab-23,^(23,24)^ which specifically recognize cancer-type PDPN in tumor tissues. For this technology, it is critical that immunogens are produced using cancer cell lines, such as LN229 glioblastoma cells, which express cancer-specific glycan-attached membrane proteins. This method can be used to develop useful mAbs against multiple membrane proteins. Using the same method, we successfully developed clone CMab-43 of IgG_2a_ subclass, which is very useful for Western blot, flow cytometry, and immunohistochemical analysis against CD133. Mouse IgG_2a_ subclass possesses several advantages over mouse IgG_1_ subclass, including antibody-dependent cellular cytotoxicity and complement-dependent cytotoxicity, and IgG_2a_ is also easy to purify using Protein-A or Protein-G.^(25,26)^

Using LN229/CD133 cells for immunization and first screening, purification of recombinant CD133 proteins is not required. This method may be applicable for developing mAbs, especially against membrane proteins that cannot be easily purified. CMab-43 was highly efficacious in Western blot analyses and produced strong staining in colon cancer cells in immunohistochemical analysis. Hence, CMab-43 will likely be an advantageous tool for the pathological identification of CD133 in many cancers.

## Supplementary Material

Supplemental data

Supplemental data

Supplemental data
