# Supplementary material for: Establishment of CMab-43, a Sensitive and Specific Anti-CD133 Monoclonal Antibody, for Immunohistochemistry
Source: Monoclon Antib Immunodiagn Immunother. 2017 Oct 1;36(5):231–5. doi: 10.1089/mab.2017.0031 (PMC6975129; doi:10.1089/mab.2017.0031)
Supplement: Supplemental data [file Supp_Fig2.pdf]

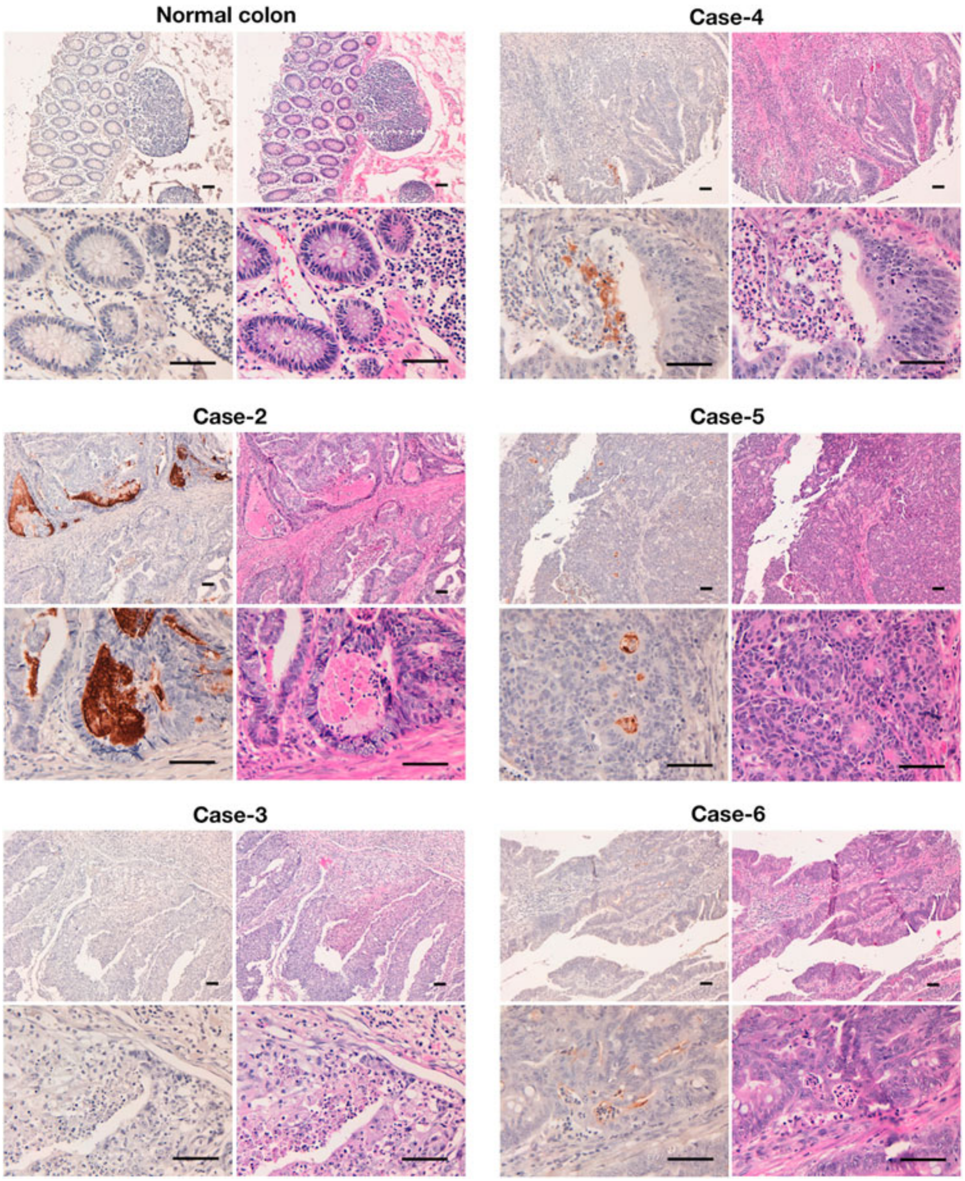

SUPPLEMENTARY FIG. S2. Immunohistochemical analysis of colon cancer by CMab-43. Sections of normal colon and colon cancer (Case Nos. 2–6) were incubated with 1 μg/mL of CMab-43, followed by the Envision+ kit. Color was developed using DAB and counterstained with hematoxylin. Serial sections were also stained using hematoxylin and eosin. Scale bar: 100 μm.
